# Broken Energy Homeostasis and Obesity Pathogenesis: The Surrounding Concepts

**DOI:** 10.3390/jcm7110453

**Published:** 2018-11-20

**Authors:** Abdelaziz Ghanemi, Mayumi Yoshioka, Jonny St-Amand

**Affiliations:** 1 Department of Molecular Medicine, Faculty of Medicine, Laval University, Quebec G1V 0A6, Canada; Abdelaziz.Ghanemi@crchudequebec.ulaval.ca; 2 Functional Genomics Laboratory, CREMI, Québec Genome Center, CHUL-CHU de Québec Research Center, Québec, Québec G1V 4G2, Canada; mayumi.yoshioka@crchudequebec.ulaval.ca

**Keywords:** obesity, pathogenesis, homeostatic mechanisms, energy misbalance, “broken energy balance”, therapy

## Abstract

Obesity represents an abnormal fat accumulation resulting from energy imbalances. It represents a disease with heavy consequences on population health and society economy due to its related morbidities and epidemic proportion. Defining and classifying obesity and its related parameters of evaluation is the first challenge toward understanding this multifactorial health problem. Therefore, within this review we report selected illustrative examples of the underlying mechanisms beyond the obesity pathogenesis which is systemic rather than limited to fat accumulation. We also discuss the gut-brain axis and hormones as the controllers of energy homeostasis and report selected impacts of obesity on the key metabolic tissues. The concepts of “broken energy balance” is detailed as the obesity starting key step. Sleep shortage and psychological factors are also reported with influences on obesity development. Importantly, describing such mechanistic pathways would allow clinicians, biologists and researchers to develop and optimize approaches and methods in terms of diagnosis, classification, clinical evaluation, treatment and prognosis of obesity.

## 1. Defining and Classifying Obesity: Fat Distribution Rather Than Fat Accumulation

The World Health Organization defines overweight and obesity as abnormal or excessive fat accumulation that presents a risk factor for diabetes, cardiovascular disease, cancer and other chronic diseases [1]. The fat accumulation towards adipogenesis and adipocytes expansion is the consequence of energy imbalances related to complex factors (genetics, environment, etc.) and results from an energy intake higher than energy expenditure. The modern obesogenic environment, characterized mainly by limited physical activity and increased caloric intake, due to both the quantity (theoretically limitless availability) and the quality (high fat and high sugar) of diet, has significantly contributed to making obesity a health problem of epidemic proportions [2] worldwide. Therefore, there is a need to better understand, define and classify obesity according to updated parameters (biological, physiological, etc.). Body mass index (BMI) is used to evaluate the obesity status at a population level [1]. Although BMI is not accurate to characterize obesity at the individual level nor a body fat measure, it is still used in the daily clinical practice due to the fact that it is simple to measure and with almost no cost. However, the clinical and biological information about the patient profile that BMI provides remain limited.

**Figure 1:**
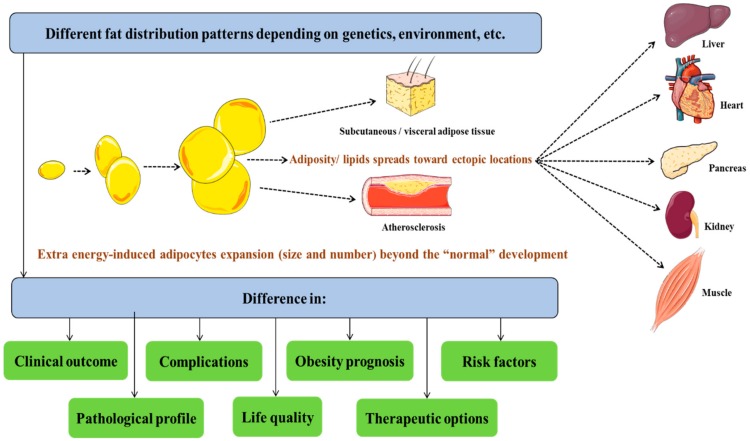
Different fat distribution patterns with diverse outcomes.

Therefore, additional parameters have been suggested to classify obesity and, more importantly, to evaluate the associated risks depending on factors beyond simple anthropometric measures. Indeed, taking into consideration more parameters, such as the hypertriglyceridemic-waist phenotype [3], waist-hip ratio (WHR) and waist circumference (WC) [4,5], has allowed a certain categorization of obese patients. However, the limits of such parameters have been shown through the history of obesity classification [6], which has encouraged researchers to continuously develop more optimized classification systems with measures that would reflect the impact that obesity has on both individual and population health. Indeed, the addition of measures, such as lipidic blood profile and fat distribution, allowed a more optimized characterization of obese patients. The first step toward establishing an accurate obesity classification system is a precise understanding of the molecular mechanisms behind its development. This requires looking at obesity as a complex metabolic misbalances in key cell types including adipocytes, hepatocytes and myocytes with a different etiologies (genetics, biological stress, unhealthy diet, etc.), leading to a variety of clinical outcomes (diabetes, hypertension, atherosclerosis, metabolic syndrome, insulin resistance, etc.) [7,8], rather than a simple fat accumulation that develops once adiposity increases beyond a “normal” level of development (Figure 1). Moreover, obesity-related metabolic patterns are not specific nor limited to some organs or tissues but rather impact all the biological systems such as endocrine functions as well as physiological homeostasis which makes obesity management treatment more challenging.

Indeed, obesity represents a systemic molecular and cellular homeostatic dysfunction. For instance, diverse illustrative examples of molecular-level impacts of obesity could be given and correlated with clinical features of obesity. Indeed, insulin resistance observed in obese patients is associated with lipid levels [9] and the palmitate lipotoxicity link with selective insulin resistance in hepatocytes put a spotlight on the lipid effects [10]. In addition, phenomena such as atherosclerosis are related to high-fat diet and levels of blood lipids [11,12]. On the other hand, reducing weight, which is mainly the loss of adiposity, leads to significant improvement in different health indicators especially cardiovascular risk factors [13,14]. Moreover, exercise and healthy diet even without weight loss still improve the cardiometabolic profile of obese individuals [15], suggesting that reducing lipid mass percentage (increasing muscle mass percentage in the case of exercise intervention) rather than the whole body weight is behind these observed beneficial effects. Therefore, the amelioration in the risk factors profile would be attributed to changes in WHR and WC, which are also two parameters that reflect both cardio-metabolic risk and fat distribution [4,5]. Thus, WHR and WC represent good parameters to optimize obesity definition, classification and the evaluation of the related risks. These observations further suggest that the underlying causes of obesity-related health disorders are lipid accumulation and distribution, rather that body weight. This is important too, precisely because some individuals have the same BMI but different body fat mass and, especially, different forms of adiposity distributions [16], indicating the importance of fat distribution types (central, ectopic, subcutaneous, etc.) (Figure 1) in pathogenesis and clinical diversity [17].

Understanding obesity through the underlying pathway changes will allow us to overcome the main challenge facing obesity studies, which includes explaining the etiologies and mechanisms of obesity linking molecular and cellular modifications to the clinical outcomes. Therefore, enabling a better understanding of this health problem is the first step toward developing efficient therapies for this medical challenge.

## 2. Gut-Brain Axis and Energy Homeostasis Hormones: Is This Neuro-Endocrine System Inefficient in Obesity?

Factors leading to or protecting from obesity, such as food intake, energy expenditure, lipids storage and glucose usage, are under the control of different neuro-endocrine systems. The gut-brain axis and metabolic hormones represent the best illustrations. Gut-brain axis refers to the signals (including gastrointestinal hormones) exchanged between selected neuroanatomical structures and the digestive system to control those factors leading to or protecting from obesity [18]. Therefore, to fully map the pathways beyond the metabolic changes, we need a description of the central mechanisms controlling energy homeostasis via gastrointestinal hormones, such as ghrelin, glucagon-like peptide 1, peptide YY 3–36, cholecystokinin and amylin, which have modified levels in obese patients compared to non-obese individuals [18]. These hormonal signals are messengers from the gastrointestinal tract, and provide the brain with a “report” about the ingested food so that the energy homeostasis centers can adjust signals toward balancing the energy metabolism (food intake and energy expenditure). However, these gastrointestinal hormones, in addition to insulin, are secreted mainly following the ingestion of carbohydrates, rather than lipids, showing that the brain may remain “blind” when lipids are ingested. This fact could be one of the significant causes of why lipids accumulate easily, since there might not be strong signals sent to the brain when lipid transit through the gastrointestinal system to limit the food intake. Moreover, dietary lipids, which are more available and more accessible than ever in the modern diet, have high caloric density and are associated with high palatability, but have a limited effect on satiety [19], which further contributes to increasing the energy intake toward developing obesity.

**Figure 2:**
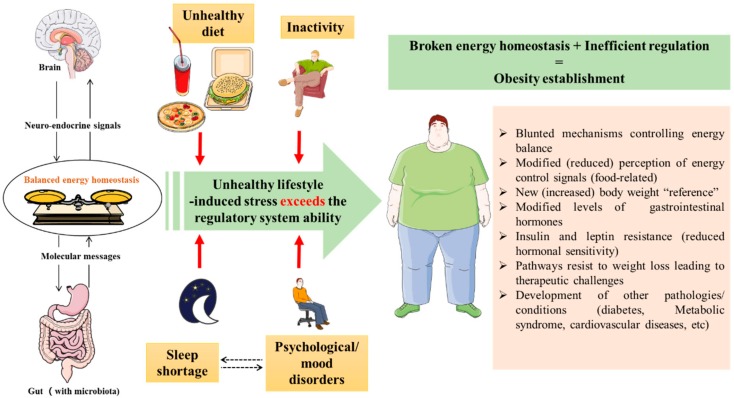
Theory of obesity establishment despite the existence of mechanisms balancing the energy homeostasis. An unhealthy lifestyle (inactivity, unhealthy diet, sleep shortage and psychological disorders) puts pressure on the energy homeostasis balance and breaks it, which leads to obesity development. Once obesity established, the mechanisms of energy balance are blunted, and the brain sets an increased body weight as a new reference, which makes correcting obesity difficult (biological pathways resist weight loss).

Importantly, further elucidation of the modifications at the gut-brain axis represents also a key concept to understanding obesity from a neuroendocrine perspective. The “deregulation” of the gut-brain axis might be the starting point for obesity pathogenesis, rather than an adaptive consequence. Indeed, once obesity is established, the mechanisms controlling energy balance that are governed by central neural networks (mainly with receptors of gastrointestinal hormones), are blunted and are not able to protect from the diet-induced obesity [20,21], nor are they able to reverse it anymore (Figure 2). Following this, the brain sets a new increased body weight “reference” and biological regulation acts toward maintaining it. This makes losing weight, or maintaining body weight after weight loss, difficult [22] due to different mechanisms. These mechanisms include metabolic slowing [22] as an adaptation ability to compensate the decreased food energy intake and/or the increased energy expenditure, along with a modification in the reward system, creating an addiction-like situation via reward circuits involving diverse neurological structures including the brain areas of the melanocortin, opioid and endocannabinoid networks in addition to the dopamine mesolimbic system [23]. Therefore, clarifying changes seen among obese patients in these brain networks would allow us to identify the related central pathways and understand the physiopathology beyond this abnormal accumulation of stored energy. An accumulation that is caused by an inefficiency of the neuro-endocrine system supposed to control energy metabolism.

Following the same line of thought, elements in food such as glutamate and fatty acids stimulate signals leading to modifications in energy metabolism that are towards weight loss [18]. Therefore, we suggest that the biological perception (and therefore the response in terms of energy balance control) of such signals would be modified in obese subjects, and with limited signaling effects. In addition to the gut-brain axis, other hormones influence the energy homeostasis. For instance, adipokines modulate various metabolic functions such as glucose and lipid metabolism, energy expenditure, insulin sensitivity and satiety [24], which makes adipokines among the key hormones in energy homeostasis. Leptin, probably the key hormone in obesity, is produced by adipose tissue [25] and controls both energy expenditure and food intake [26]. The production of this hormone correlates with the body fat mass which highlight it as modified in obesity and thus, worth exploring for a potential “molecular description” of obesity at the central level as well (central control, rather that direct peripheral metabolic effects).

Importantly, since a neuro-endocrine system that controls energy homeostasis exists, how can some individuals still develop obesity? This is probably explained by the limits of resistance of this system. Although this system tends to balance energy expenditure and intake, putting “biological pressure” on this system via an unhealthy lifestyle such as high-fat diet and inactivity will overcome the efficiency of this system and exceeds its regulatory ability. Once the energy homeostatic balance is “broken”, the neuro-endocrine system will fail to establish energy balance due to the inefficiency of this system (Figure 2). Importantly, the role played by gut microbiota within the context of obesity and the gut-brain axis [27] makes it worth exploring, as well. However, at this stage, it seems essential to ask an important question: is this neuro-endocrine system equilibrium broken after obesity develops, or does obesity represent a consequence of the loss of the efficiency of this system? Answering this question would be a major step towards understanding obesity pathogenesis, and therefore, how it should be treated.

## 3. Metabolic Tissues Undergo the Energy Misbalance and the “Modified Paradigm” of Signaling Molecules in Obesity

Biology gave humans the ability to store energy as a tool to face situations of hunger and food shortage. From an evolutionary viewpoint, and compared to modern societies, humans who lived decades ago did not have limitless access to food, nor did they follow the modern unhealthy lifestyle [28]. Thus, the tissues and organs under the modern obesogenic environment, which represents a biological pressure, undergo changes that could be adaptive to this imbalanced energy homeostasis.

Adipose tissue has the ability to store energy, but once the balance of the gut-brain axis is broken (Figure 2), the expansion of adipose tissue both in size and cell number becomes important, obesity is established and adiposity spreads toward ectopic locations [16], which results in different fat distribution patterns (Figure 1). The starting point of metabolic consequences of obesity is mainly due to increased fat storage with the visceral adiposity as the most deleterious form. For instance, a study aiming to map the visceral adipose tissue metabolism pattern in obese subjects has shown increased oxidative stress and markers and markers of elevated glucose levels, in addition to elevated levels of plasmalogens and changes in various lipidic molecules (including glycerol-phosphorylcholine, ceramides and sphingolipids) [29]. Such observations fit with and explain some of the clinical aspects seen among obese patients or epidemiologically linked to obesity. As an illustration, the increase in oxidative stress-produced free radicals correlates with the epidemiological links between obesity and some cancers [30,31,32].

In addition, variations in various lipidic molecule patterns observed in obese patients show that the problems associated with lipids in obese patients is not limited to an accumulation in adipose tissue, but are rather exported to other tissues and systems (Figure 1) such as the liver (non-alcoholic steatohepatitis) [33], blood vessels walls (atherosclerosis) [34], and muscles (insulin resistance) [35]. This illustrates the systemic character of obesity consequences. Within this context, the known effects of hormones like insulin and leptin on the metabolism of lipids and glucose/glycogen act mainly on the muscle and adipose tissue [36]. Changes seen in these two key hormonal systems in obese subjects allow us to predict the impact of obesity on the main metabolic organs as a consequence of modified insulin sensitivity for example, especially with the interactions between leptin and insulin [37] and the distribution of leptin receptors [38]. These lead to important metabolic imbalances in lipid and glucose metabolism so that these two energy substrates become “biotoxic” rather than just biofuels. Indeed, diabetes complications illustrate the consequences of a deregulated glucose metabolism and cardiovascular disease points to those of lipid metabolism imbalances.

In obesity, we have increased adiposity with greater adipocyte size and number, leading to a higher leptin concentration, but without reduced food intake (leptin resistance [39]), which is an example of the consequences of altering the neuro-endocrine system controlling energy homeostasis. Moreover, the increased body weight of obese patients suggests an increased need for muscle to carry extra weight, which would require more energy usage that could also be affected by insulin resistance (resulting from unhealthy life style [40]) and deregulation of the metabolism of both glucose and lipids. Metabolic syndrome [41] could be seen as the most significant manifestation of these metabolic disorders, where systems and tissues become metabolically unhealthy, with insulin signaling being at the center of the syndrome [7]. Importantly, the implication of both leptin and insulin in diverse homeostatic functions (not only energy metabolism) [39,42] makes the development of insulin and leptin resistance a physiological problem beyond energy metabolism. Furthermore, since brown adipose tissue (BAT) is not only under the influence of the sympathetic nervous system but is also controlled by factors such as adipokines and metabolites [43], the balance and metabolic properties of this adipose tissue may be modified in obese patients following the hormonal deregulation as well.

These selected examples highlight some of the effects of obesity on key metabolic tissues (adipose tissue, liver and muscle), placing obesity among the top metabolic disorders and further support the need for a “metabolic classification” for obesity. Moreover, our illustrations highlight the concept of the “broken homeostatic system” (Figure 2), within which signaling molecules properties and metabolites affect the physiological systems differentially during obesity compared to a healthy status. Such a concept of a “modified paradigm” of hormones and signaling molecule effects on metabolic functions of obese individuals is extremely important, since it indicates how the same hormone, for instance, could have different properties (for example insulin resistance or sensibility) depending on whether it is under physiological (healthy) or pathological (obesity) conditions, and could therefore explain possible “contradictory” results of studies comparing healthy individuals with obese patients beyond which we can find that gut-brain axis hormone levels in obese patients are different from those in non-obese individuals [18].

## 4. Beyond the Energy Balance: Sleep Shortage and Psychological Effects as Examples of “Non-Caloric Factors”

**Figure 3:**
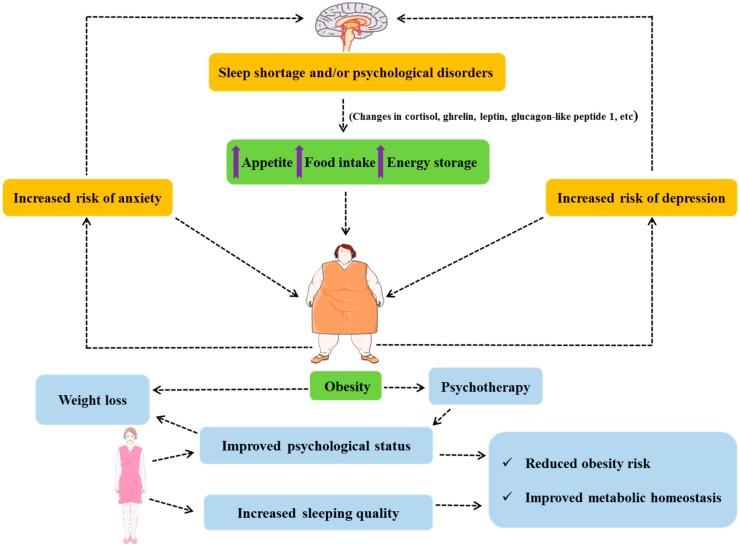
Sleeping, psychology and obesity.

Obesity cannot be reduced to a biochemical balance or a mathematical model of energy homeostasis. It is, indeed, a complex consequence with a variety of influencing factors, including “non-caloric” representing factors other than food intake and physical activity. For instance, sleep shortage has been linked to obesity [44,45,46], and the literature also reports how circadian rhythms regulate diverse metabolic functions [47]. The studies of Spiegel et al. indicated lower glucose tolerance, decreased leptinemia, increased cortisol and ghrelin plasma concentrations, and higher hunger and appetite in cases of sleep deprivation [48,49]. Additionally, sleep restriction, via a variety of pathways, also increases insulin resistance with risk of diabetes and modifies appetite-related hormones such as glucagon-like peptide 1 and leptin [50]. These alterations allow us to map a metabolic link between restricted sleep duration and obesity via selected hormonal (leptin, cortisol, glucagon-like peptide 1 and ghrelin) functions and the metabolism of lipids and carbohydrates. Indeed, such biological changes toward increased food intake and energy storage lead to fat accumulation (Figure 3). The fact of staying awake could send “wrong signals” to the brain to indicate the need for staying active, and thus a need for energy, which leads to an increased appetite combined with energy storage (fat accumulation) as an “adaptation” to this energy need which is, in fact, due to the control mediated by hormones (cortisol, ghrelin, etc.) that are also deregulated under such sleep shortage conditions. Thus, a regulated circadian rhythm should remain a part of a healthy life style, as well as an approach to obesity management and metabolism regulation [51]. Additionally, it is worth mentioning that both psychological outcomes and sleeping quality are improved following weight loss [52,53], which would reduce obesity risk and improve metabolic profile. This also indicates that not only do sleeping and psychological status affect obesity development, but obesity also has an impact on these two parameters, as well (Figure 3).

On the other hand, different studies have also linked psychological disorders and mental health problems such as depression and anxiety [54] to obesity [55,56,57,58] (Figure 3). Such problems could either be directly related to obesity or rather be a consequence of how society—Including healthcare professionals—Behaves toward obese patients [59], and thus could be described as a sociopsychological consequence. In both cases, assistance could be required in the form of psychotherapy, social help or even economic assistance for obese patients. Moreover, psychotherapy [60] will not only assist the obese patients in dealing with the psychological consequences related to obesity, but it could also further stimulate them and strengthen their will to adhere to weight loss programs [61]—Via sports psychology, for instance [62]—To changes behavior [63], which is key to initiating obesity therapy.

Within this context, it would seem acceptable to assume that sleep shortage and psychological factors contribute to the status of a “broken homeostatic system” described previously (Figure 2), leading to inefficient energy metabolism control. Moreover, sleep quality and psychological factors are two concepts that are often associated with each other. Thus, having a problem with either would affect the other and increase the probability of developing obesity or worsen the prognosis when obesity or overweight are already present. Moreover, obesity association with mood disorders (depression and anxiety risk) and cognitive deficiency (in learning and memory) [27] would create a vicious circle. Importantly, the possible interactions between the different neuronal networks involved in energy balance, sleep control and psychologic (and psychiatric) regulation would not only explain the effects of sleep shortage and psychology, as “non-caloric factors”, on obesity but also require an increased pharmacovigilance for pharmacotherapies targeting any of these systems to prevent the effects on the other neuronal networks [64].

## 5. Toward an Optimized Obesity Management

The BMI and different anthropometric measures represent the most commonly used parameters to classify individuals as overweight, obese or “normal”-weight. However, evaluating the biological disorders (glucose metabolism disorders, lipid accumulation, etc.) and mechanistic imbalances seen in obese patients at various levels (hormonal, metabolisms, psychology, signal resistance, etc.) should be used as supplementary elements in diagnosis and classification. This description would explain the underlying mechanistic origins of the clinical manifestations of obesity such as hyperlipidemia and hyperglycemia, rather than just report them. In addition, establishing a mechanistic link between the pathogenesis of obesity and molecular biochemical variations (hormonal levels, glycemia, triglycerides, fatty acids, etc.) could make it possible to either add or exclude biological variables from the classification parameters of obesity, diagnosis and therapeutic targets. Also, putting a spotlight on the molecular mechanisms of obesity may strengthen the available diagnostic tools by adding new biological parameters to the standard measure of BMI. Moreover, it clarifies the links between obesity and other metabolic-related diseases such as diabetes, inflammation and metabolic syndrome [41].

Importantly, these same parameters could be used as tools to establish prognosis, follow the clinical evolution and evaluate obesity therapies during laboratory research as well as clinical trials. Measuring such parameters will also represent strategic tools for prognosis and evaluation of the therapeutic approaches, although it might have a high cost. For instance, measuring different intestinal hormone levels may be a good indicator of the gut-brain axis interaction acting in terms of energy storage, food intake and appetite.

The mapping of such mechanistic pathways in metabolic and energy homeostasis in overweight and obese patients, leading to obesity-related disorders, would allow a more optimized definition and classification of obesity by taking into consideration more parameters among those described within this review. Considering these mechanistic changes and their clinical consequences in obese patients makes the pathways related to them potential therapeutic targets for obesity or at least for one of the symptoms or even other diseases that involve one or more of the metabolic changes seen in obesity. Indeed, exploring factors related to the metabolism of energy and appetite control, such as hormones of the neuro-endocrine system, represents an emerging path for the treatment of obesity [18] and the related disease, including adipokine-based strategies [65]. More importantly, the existence of an adrenergic-independent possibility to activate the BAT represents strong hope of losing weight via increased energy dissipation [43]. This approach exploits the high capacity of the BAT to uncouple the oxidative phosphorylation from the production of adenosine triphosphate (ATP) and dissipate energy in heat form rather than synthesizing ATP, which makes BAT a biological calory-burning engine. This principle could be combined with the white adipose tissue (WAT) beiging promotion [65] to both reduce WAT and increase energy expenditure.

On the other hand, the complex interactions of the homeostasis control centers and the various implicated neurotransmitters could allow us to consider obesity as a consequence of a deregulation at the nervous system and thus similar to a neurological disorder. Such an approach could be a starting point towards a more sophisticated neuropharmacological treatment of obesity that targets the neurological structures and neurotransmitters involved in energy homeostasis control. Moreover, the reward system underlying hedonic stimulation could be targeted, via an approach that mimics drug addiction therapies. However, the side effects and the related pharmacovigilance would be a challenge due to the complexity of the neuroendocrine system. Following the same line of thought, and due to the strong hormonal component in obesity, hormonal pathways (such as leptin and insulin-related) still deserve further investigations to identify novel molecular targets within the obesity-related pathways of these hormones.

Importantly, studying the metabolic effects of obesity might make some diseases considered as chronic (such as type 2 diabetes) “reversible” [66]. Indeed, since type 2 diabetes results in part from insulin resistance, treating obesity would increase insulin sensitivity and lead to a remission of type 2 diabetes [67], especially with the emerging concept of adipose tissue targeting to treat obesity-associated diabetes [65] with increased clinical hopes.

Moreover, the ability of the adipose tissue to store elements such as persistent organic pollutants and the important biological functions governed by adipose tissue (metabolic, protective, etc.) [68] reminds us about the importance of this tissue and, more importantly, increases the pharmacovigilance associated with obesity therapies and adverse effects including those related to the liberation of organic pollutants stored in the adipocytes. Such side effects demonstrate that further development of new therapeutic approaches and combining therapies with those existing is required in order to optimize them.

New research strategies, such as functional genomics and the development of novel animal models [69], would further clarify the molecular pathogenesis of obesity and map the links with the clinical aspects along with the therapeutic consequences. Importantly, the clinical consequences of developing obesity represent a bigger challenge than obesity itself. Therefore, the objective of clinicians and health care professional should not be exclusively weight loss but rather assisting the patients to establish a healthy lifestyle that balances diet, physical activity, sleep cycle and psychological status. Such an approach would not only lead to weight loss and fat mass reduction, but also improve the clinical factors related to or affected by obesity such as insulin resistance, lipid metabolism disorders, cardiorespiratory fitness, cardiometabolic conditions and chronic inflammation. Furthermore, this same approach of balancing life style is beneficial even without weight loss, since many individuals who follow specific diets or exercises do not lose weight. Thus, weight loss should be seen as one single result among the benefits of a healthy life style prescribed for obese individuals rather than the unique objective especially that the risks are about the location and the distribution of the adipose tissue (mainly visceral adipose tissue) rather that the adiposity that could be subcutaneous for example, reflecting the need to redefine obesity [70], how we assess it, treat it and establish the prognostic of the obese patients in order to further link the underlying mechanisms to the clinical outcomes and prognosis.
